# A Rare Case of Spontaneous Idiopathic Pneumoperitoneum Presenting as Abdominal Pain

**DOI:** 10.7759/cureus.15158

**Published:** 2021-05-21

**Authors:** Jagmeet S Grewal, Savannah Mayer, Jennifer Beaty, Dominic Formaro

**Affiliations:** 1 Medicine, Des Moines University, Des Moines, USA; 2 Surgery, Des Moines University, Des Moines, USA; 3 Surgery, UnityPoint Health Methodist, Des Moines, USA

**Keywords:** idiopathic spontaneous pneumoperitoneum, spontaneous pneumoperitoneum, pneumoperitoneum, conservative treatment, laparoscopy, free air under the diaphragm

## Abstract

Idiopathic spontaneous pneumoperitoneum is caused by free air in the peritoneum when no established cause has been diagnosed. We present the case of a 61-year-old male with idiopathic spontaneous pneumoperitoneum, which started as abrupt abdominal pain. He described burning abdominal pain radiating to his right shoulder and endorsed symptoms of nausea, abdominal bloating, and heartburn but denied fever, chills, or vomiting. Chest radiograph and computed tomography demonstrated massive amounts of free air under the diaphragm, concluding an extensive pneumoperitoneum. He was diagnosed by standard imaging modalities and then underwent diagnostic laparoscopy, which did not reveal any areas of perforation. Subsequently, the patient had an uncomplicated recovery. The complexity of diagnosis and treatment has made it difficult for surgeons to treat spontaneous pneumoperitoneum patients.

## Introduction

Pneumoperitoneum is defined as free air within the peritoneal cavity with a majority of causes resulting from intra-abdominal viscus perforation [1]. Peptic ulcer disease is considered the most common cause of pneumoperitoneum, with perforation of either the gastric mucosa or duodenum. Furthermore, perforation of any part of the bowel may lead to the formation of pneumoperitoneum [2]. Patients usually present with signs of peritonitis (fever and/or peritoneal irritation) with evidence of intra-abdominal free air seen on imaging. The presence of pneumoperitoneum usually indicates emergent laparotomy, although surgery is not required when there is no evidence of visceral perforation [3].

When there is no evidence of visceral perforation and indication for surgery, the diagnosis of exclusion is called spontaneous pneumoperitoneum (SP) or "non-surgical" pneumoperitoneum. The causes of SP are relatively complex but can involve intrathoracic, intra-abdominal, gynecologic, or iatrogenic causes [1]. The diagnosis and treatment of conventional pneumoperitoneum involve diagnostic laparoscopy/laparotomy, but SP patients may undergo unnecessary surgery since SP can be treated by conservative measures [2,3]. The complexity of diagnosis and treatment has made it difficult for surgeons to treat SP patients. In this case report, we present the case of a 61-year-old male with a massive SP presenting with abdominal pain. 

## Case presentation

**Figure 1:**
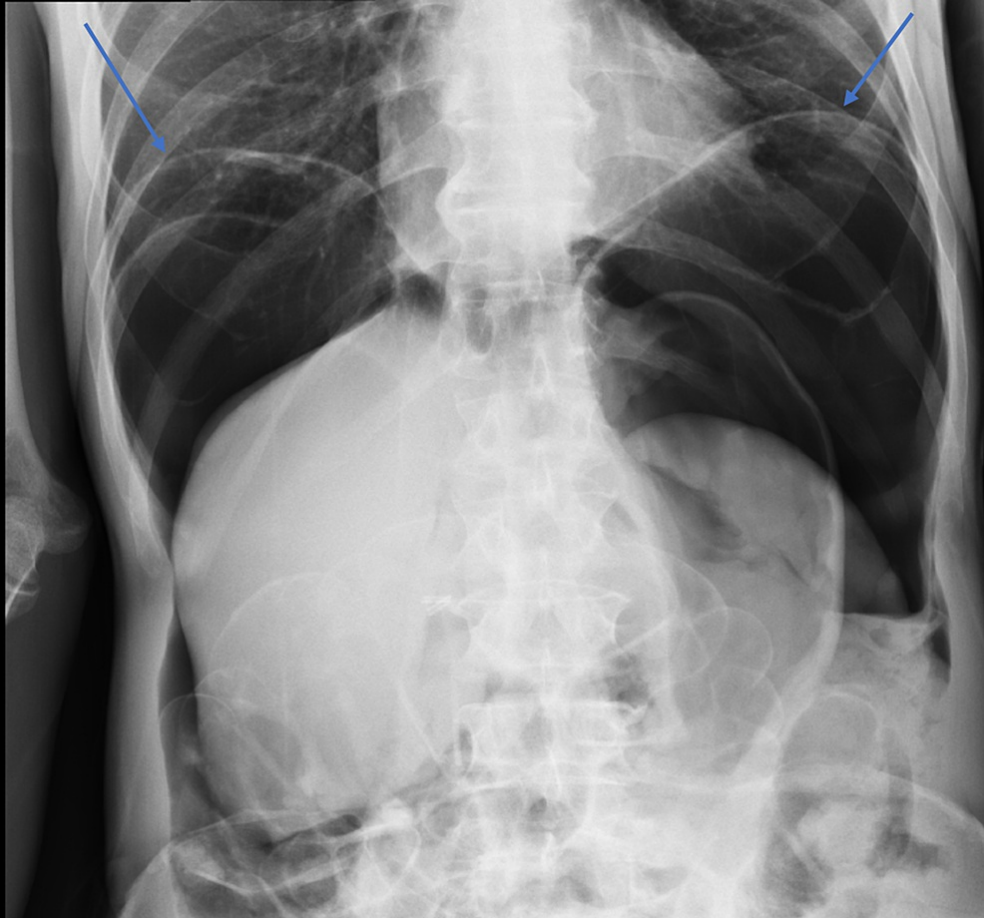
Chest radiograph Extensive pneumoperitoneum, bilateral free intraperitoneal air (blue arrows), noted throughout the abdomen.

A 61-year-old male patient with a past surgical history of appendectomy, cholecystectomy, and Nissen fundoplication (refractory to gastroesophageal reflux disease) presented to urgent care with complaints of abdominal pain that started abruptly. He described burning abdominal pain radiating to his right shoulder and endorsed symptoms of nausea, abdominal bloating, and heartburn but denied vomiting. The patient confirmed flatus and bowel movement since the time of onset. He reported having a recent left rotator cuff repair, without complication, and denied taking nonsteroidal anti-inflammatory medications in his postoperative period. He was given a treatment of “GI cocktail” with 30 mg of toradol intramuscularly. Subsequent X-ray was obtained, which demonstrated an extensive pneumoperitoneum. Also, diffuse mildly conspicuous gas-filled loops of large and small bowel were noted throughout the abdomen with no convincing portal venous gas (Figure 1). Patient was transferred to the emergency department for further workup. 

**Figure 2:**
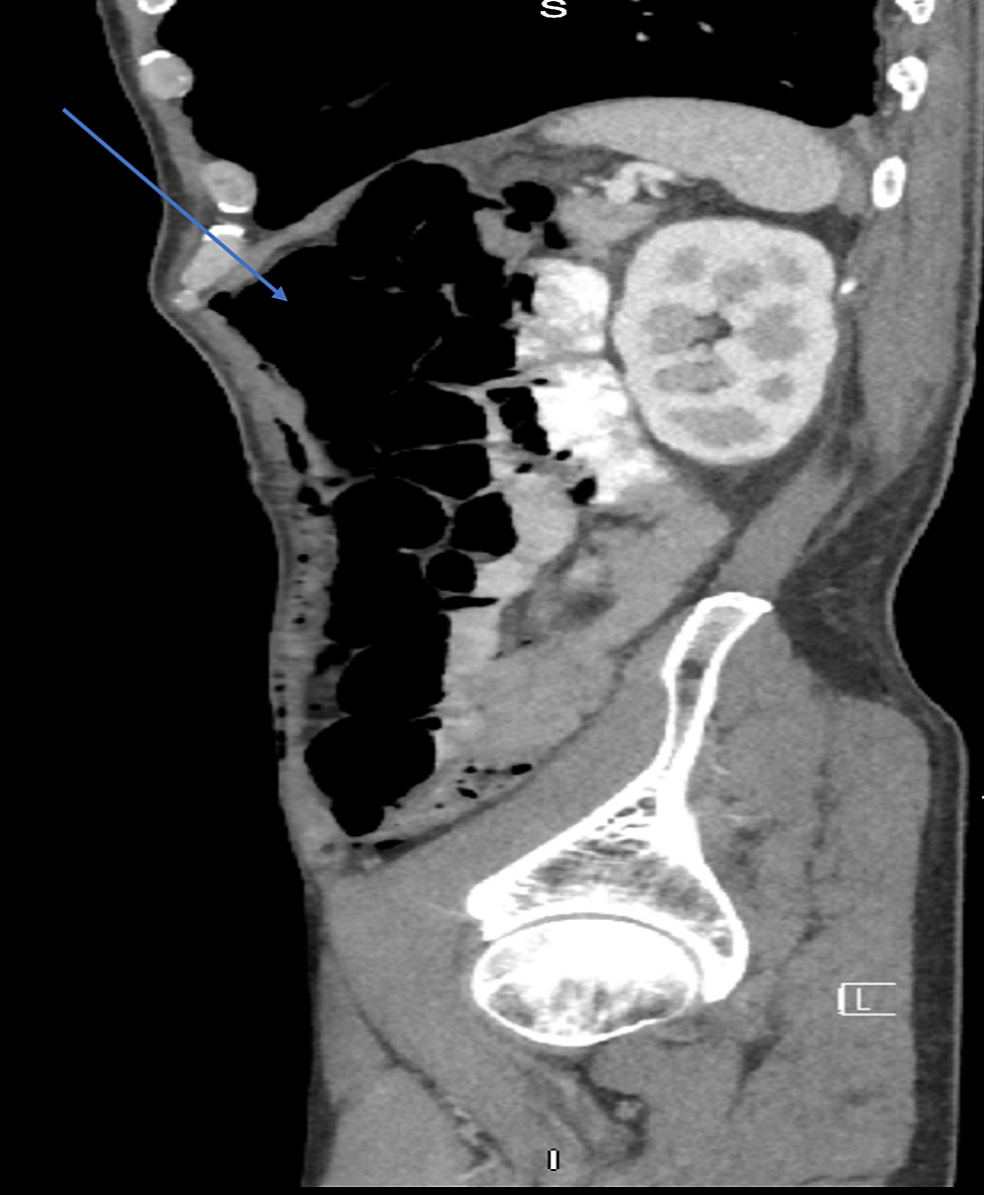
Sagittal CT scan Significant pneumoperitoneum demonstrated by free air in the peritoneal cavity (blue arrow).

In the emergency department, the patient appeared to be in progressive distress and was found to have significant abdominal tenderness in all four quadrants, worse in the epigastrium. His focused abdominal examination was significant for guarding, distention, and tympany to percussion. Subsequently, general surgery was consulted for further management. CT was ordered (Figure 2). Further history revealed a remote history of peptic ulcer disease and negative history of colon complications or constipation. He reported normal bowel movements throughout the week. The patient was taken to the operating room for a diagnostic laparoscopy to evaluate for upper abdominal perforation with possible bowel resection with an ostomy. 

**Figure 3:**
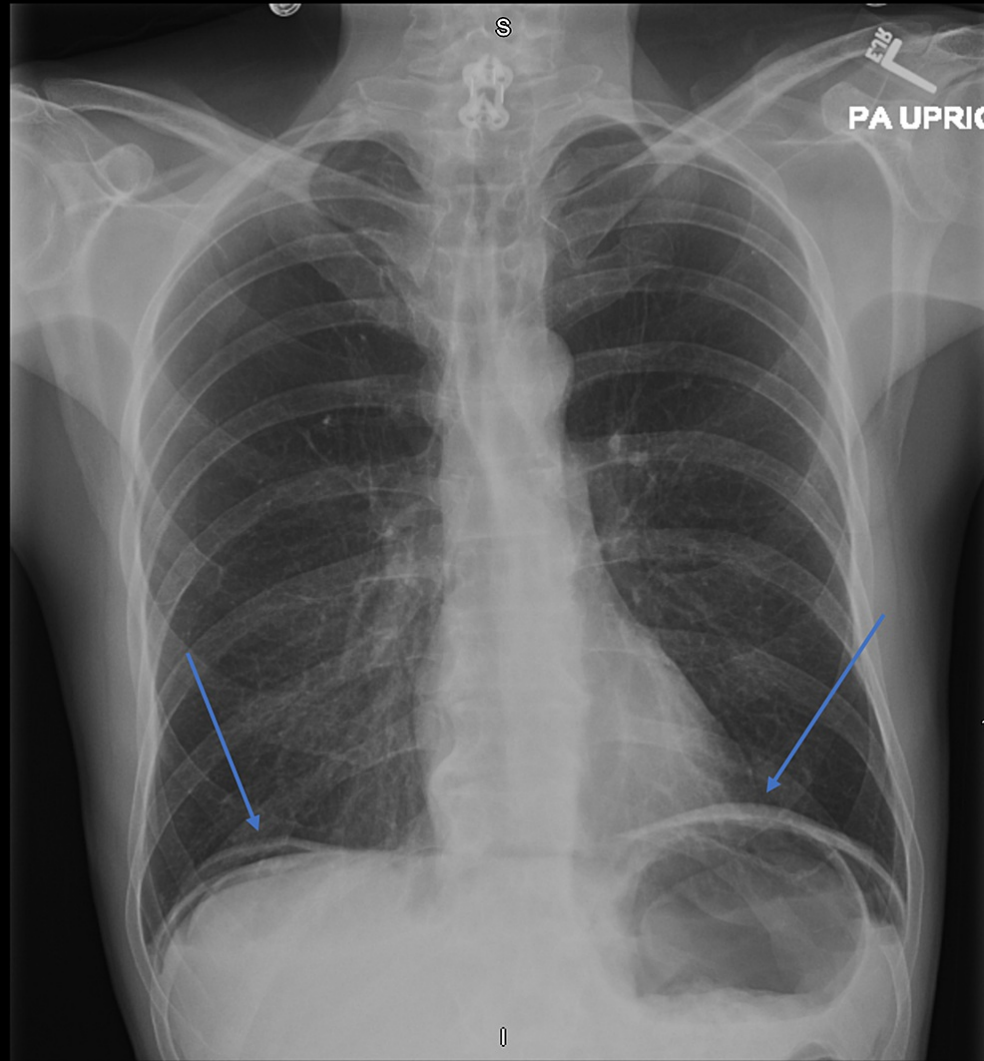
Chest radiograph Minimal radiological evidence of extensive pneumoperitoneum (blue arrows).

**Figure 4:**
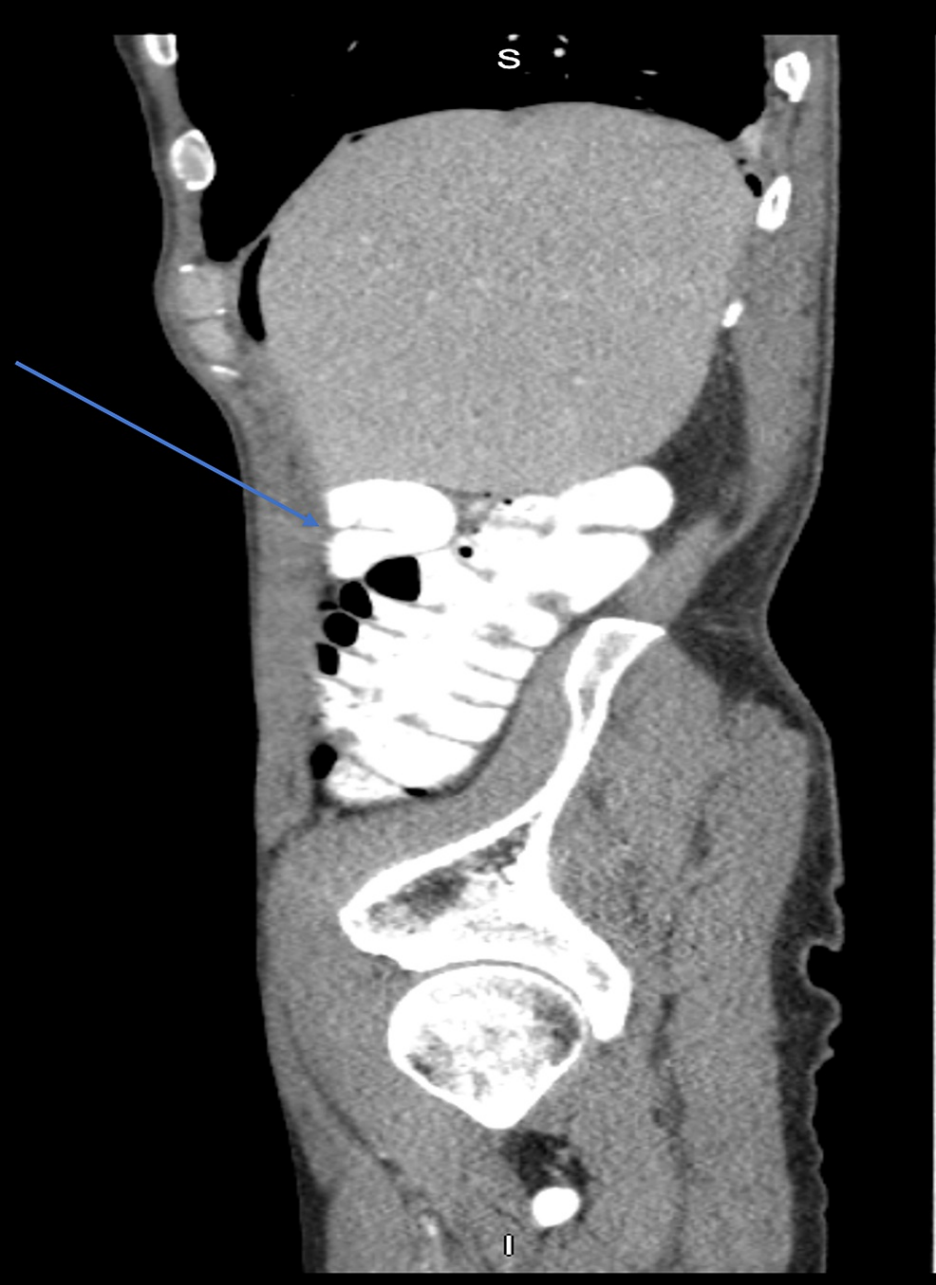
Sagittal CT scan No evidence of free air in the peritoneal cavity, and pneumoperitoneum has resolved (blue arrow).

The laparoscopy showed no evidence of contamination, peritonitis, or perforation throughout the abdomen and the remaining gas was suctioned from the abdomen before closing. The patient’s symptoms resolved over the course of a couple of days in postoperative recovery. X-ray and CT imaging were repeated prior to discharge and demonstrated significant improvement of the pneumoperitoneum, with only a small volume of gas remaining (Figures 3, 4). An esophagogastroduodenoscopy was also performed, which was unremarkable and ruled out ulceration as a possible cause. After ruling out known causes of pneumoperitoneum, it was concluded that the patient had experienced an idiopathic SP. At his follow-up visit, the patient reported an uncomplicated recovery and denied any recurrence of symptoms.

## Discussion

Pneumoperitoneum with perforated intra-abdominal viscus accounts for 90% of all causes, with the remaining cases labeled as idiopathic SP. Risk factors for perforations include diverticulosis, obstruction, advanced age, and existing weaknesses in the colonic wall that have resulted from inflammatory bowel disease, infection, or malignancy [2-4]. Patients with pneumoperitoneum from visceral perforation present with nausea, vomiting, fever, and peritonitis, which requires immediate surgical intervention. The complexities lie with patients with minimal symptomology and unclear sources. Patients can have a visceral perforation, but may appear to be stable [4,5]. These patients can be treated with either diagnostic laparoscopy/laparotomy or close observation. In patients with minimal symptomology and SP, a high index of suspicion and careful history and complete physical examination are required to determine the source of free air [6].

The vast majority of pneumoperitoneums are caused by retained postoperative air, but other sources of free air include mechanical ventilation, cardiopulmonary resuscitation, and pneumothorax [5,6]. Excessive quantities of gas contained in the abdominal cavity with progressive increase of intra-abdominal pressure can be labeled as tension pneumoperitoneum or abdominal tamponade. Furthermore, it is hypothesized that there are five sources from which air can enter the peritoneum: oropharyngeal surgery, intrathoracic source, intra-abdominal source, extra-abdominal source, and idiopathic [7]. Following oropharyngeal surgery, air can traverse via the mediastinal route resulting in air into the peritoneum. Also, barotrauma from significant positive pressure ventilation into the thoracic cavity may cause air passage into the subcutaneous space and ultimately into the peritoneum. Interestingly, ruptured intramural gas-filled cysts in the gastrointestinal tract, caused by pneumatosis cystoides intestinalis, can cause pneumoperitoneum. Moreover, free air can enter the thorax and into the peritoneal cavity through two different routes. First, the direct passage from the pleural cavity through a diaphragmatic defect either congenital or pathological. Second, through the mediastinum and along the great vessels leading from the chest to the abdomen, entering the intestinal mesentery and finally rupturing into the peritoneal cavity [7-9]. Furthermore, in females, the genital route is a possible port of entry into the abdomen caused by vaginal insufflation due to sexual intercourse. Finally, there are cases where the underlying cause cannot be determined even with a thorough examination, which would be classified as idiopathic [9-11].

The diagnosis of SP requires a detailed history and physical examination, with further imaging including chest radiograph and CT. Plain chest or abdominal radiograph as well as CT will show free subdiaphragmatic air. If the patient has a perforation, CT findings predict the site of gastrointestinal tract perforation with 86% accuracy [2]. Also, one can consider a pneumogastrogram, which is a cost-effective and safe method to exclude visceral perforation. It has been shown that pneumogastrograms can increase the diagnostic yield of pneumoperitoneum from 66% following plain X-ray examination to 91% and is free of complications [2,12]. These methods are useful to distinguish surgical vs nonsurgical pneumoperitoneum, so an unnecessary laparotomy can be avoided. Even with multiple imaging modalities, most SP patients undergo exploratory laparotomy. It is reported that 28% of patients with misleading pneumoperitoneum findings were subjected to surgical interventions, which might not have been necessary [13]. Furthermore, there have been cases of patients undergoing exploratory surgery without any evidence of perforated viscus, suggesting that surgeons have difficulty diagnosing and indicating the correct course of treatment [13,14].

Conservative management should be the mainstay for patients with SP with minimal symptomology, with some studies suggesting even so when signs and symptoms of peritonitis are present [9,15]. No conclusive conservative management protocol has been published, but the consensus of conservative treatment is in parallel to a perforated peptic ulcer since it is the most common cause [2]. Patients with pneumoperitoneum localized to the upper abdomen, who are hemodynamically stable and have minimal to mild symptoms, should be treated with anti-ulcer medication, such as H2 blockers and proton-pump inhibitors [16,17]. It is imperative that patients still be under frequent and careful clinical monitoring. In doubtful situations, laparoscopic exploration can be performed as a good alternative to laparotomy [18].

## Conclusions

We presented a case of idiopathic SP in a 61-year-old male who presented with abrupt abdominal pain. He underwent diagnostic laparoscopy after multiple imaging modalities, but no evidence of visceral perforation was noted. The complexity of diagnosis and treatment has made it difficult for surgeons to treat SP patients. Of note, patients can undergo laparoscopic exploration as an alternative to laparotomy to minimize postoperative complications. Our patient reported an uncomplicated recovery and denied any recurrence of symptoms after his diagnostic laparoscopy. 
